# An analysis of associations of having children with smoking prevalence and intensity using nationally representative survey data in Japan

**DOI:** 10.1186/s12889-026-26817-3

**Published:** 2026-02-27

**Authors:** Tasuku Okui

**Affiliations:** Medical Information Center, Kyushu University Hospital, 3-1-1 Maidashi, Higashi-ku, Fukuoka city, Fukuoka prefecture 812-8582 Japan

**Keywords:** Japan, Smoking, Children, Marital status

## Abstract

**Background:**

In Japan, the association with smoking status and having children after infancy has not been investigated. In this study, we examined the associations of having children with smoking prevalence and intensity using nationally representative survey data in Japan.

**Methods:**

A cross-sectional study was conducted, and data on the Comprehensive Survey of Living Conditions in 2010, 2013, 2016, and 2019 were used. Having children aged 0–4 years, having children aged 5–9 years, having children aged 10–14 years, having children aged 15–19 years, and the number of children were used as the indicators of child presence. The analysis was conducted by sex and marital status. We used a modified Poisson regression model to reveal the association between having children and smoking prevalence, and an ordinal logistic regression was used to investigate the association with the number of cigarettes smoked per day. Age group, household income, educational attainment, marital status, residential type, employment status, and year were used as other characteristics.

**Results:**

Data from 18,261 people were analyzed. Having children aged 0–4 years was associated with lower smoking prevalence or fewer cigarettes smoked per day both in women and men, while no significant association was observed in unmarried women. In addition, having children aged 5–9 years, 10–14 years, or 15–19 years was not significantly associated with a lower smoking prevalence and fewer cigarettes smoked per day in women and men, whereas having children aged 5–9 years and having children aged 15–19 years were significantly associated with a higher smoking prevalence in married men. Moreover, a higher number of children was significantly associated with lower smoking prevalence in women, while having ≥ 3 children was significantly associated with higher smoking prevalence in married men.

**Conclusions:**

The associations of having children with smoking prevalence and intensity differed by child age, the number of children, parental sex, and marital status. Particularly, it was suggested that smoking cessation efforts are required among parents having children aged ≥ 5 years.

**Supplementary Information:**

The online version contains supplementary material available at 10.1186/s12889-026-26817-3.

## Background

Smoking is a risk factor for multiple types of diseases [1, 2], and secondhand smoke is also a risk factor [3, 4]. Children are vulnerable to secondhand smoke, and associations of parental smoking with adverse outcomes, including obesity, cognitive skills, and behavioral problems, have been demonstrated [5–7]. Evidence indicates that secondhand smoke exposure in childhood was associated with a higher risk of obesity and coronary heart disease in adulthood [8, 9]. In another study, secondhand smoke exposure was associated with higher smoking prevalence of adolescents [10], and parental smoking status was also shown to affect the smoking prevalence of children [11]. Therefore, it is important for parents to refrain from smoking for the health of their children.

Having children is known to be associated with smoking status, as some studies have demonstrated that having children is linked to a lower smoking prevalence [12, 13]. Another study showed that the association varied with the age of the child and the marital status of women [14], while there is a study showing no association between having children and smoking status [15]. Moreover, associations of the number of children with smoking status have been investigated in some studies [16, 17]. In Japan, there are some studies investigating an association between childbirth and changes in smoking status, and it is known that the prevalence of smoking in mothers decreased during pregnancy and bounced back 18 months after delivery [12, 18]. In addition, a study investigated factors associated with post-parturition smoking relapse among former-smoker women [19]. However, to the best of our knowledge, the association of smoking status with having children after infancy has not been investigated, despite the relevance of investigating whether the presence of school-aged or teenage children is associated with smoking prevalence. Also, to our knowledge, it has not been investigated whether the association between smoking status and having children varies with marital status and the number of children. By investigating those, we could better understand the target population to whom recommendations of smoking cessation are particularly needed.

Therefore, in this study, we investigated the associations of having children with smoking prevalence and intensity using national survey data in Japan.

## Methods

### Data

A cross-sectionals study was conducted. The data on the Comprehensive Survey of Living Conditions in 2010, 2013, 2016, and 2019 were used in the analysis. The survey aimed to investigate the current statuses of the daily lives of Japanese citizens, and information on household, income, savings, nursing care, and health was surveyed. The survey was carried out by the Ministry of Health, Labour and Welfare, and the health-related survey is conducted every 3 years. We used data including the survey on household income. Districts were randomly selected based on stratified random sampling in each year, and all households within the districts were targets of the survey. In the survey on income, questionnaires were sent to 35,971, 36,419, 34,286, 32,529 households in 2010, 2013, 2016, and 2019, respectively, and the response rates were 75.7%, 74.4%, 73.7%, and 70.7%, respectively [20]. The anonymous data, which were the resampled data of the survey, were provided by the Ministry. In the anonymous data, households with ≥ 8 members, single-father households, households with ≥ 2 members needing nursing care (members who were aged ≥ 6 years and who needed care because of disability or decline in physical functions), households with ≥ 2 members with certificates of nursing care, households with parents and children whose age differences were either too large or too small, and households with ≥ 4 members in the same age range were excluded by the Ministry for anonymity of the data [21].

Data on household, sex, 5-year age group, household income, educational attainment, marital status, residential type, employment status, smoking status, and hospitalization were analyzed. The questionnaire items used in this study were consistent throughout the years. According to marital status, study participants were classified into married, never-married, and widowed/divorced. According to educational attainment, they were classified into less than high school, high school, professional training college, technical college/junior college, and university, and those currently enrolled in school. According to employment status, they were categorized into regular workers, non-regular workers, self-employed workers, other workers, and unemployed persons. By residence type, participants were categorized into owning a house, rental/employment housing, and lodging/others. Household income was categorized into quantiles. Regarding the smoking status, the questionnaire options were “smoking every day,” “smoking sometimes,” “formerly smoked but has not smoked for at least a month,” and “not smoking.” “Smoking every day” and “smoking sometimes” were classified as smokers, and the others were classified as nonsmokers. In addition to the current smoking status, the number of cigarettes smoked per day was asked of the smokers in the questionnaire, and we used it as an outcome variable in addition to smoking prevalence. The options for the number of cigarettes smoked per day were “≤10 cigarettes,” “11–20 cigarettes,” “21–30 cigarettes,” and “≥31 cigarettes.” Because the number of persons with “≥31 cigarettes” was relatively small, particularly in women, “21–30 cigarettes” and “≥31 cigarettes” were combined into one group. Therefore, the number of cigarettes smoked per day was used as an ordinal categorical variable consisting of 0 (nonsmoker), “≤10 cigarettes,” “11–20 cigarettes,” and “≥21 cigarettes.”

Information on family relationships in a household was used to identify the status of having children for each person. The family relationship with the household head was available for each household member, and household heads, their spouses, their children, or spouses of their children were used in the analysis because it was possible to identify the status of having children for those persons. The households of those persons included not only two-generation households but also three-generation households, and it was also possible to identify grandchildren of the household head in the three-generation households. If an unmarried person and married persons—offspring of the household head or their spouse—lived in the same household, the grandchildren of the household head were judged as the children of married persons in the analysis. If multiple unmarried persons—unmarried children of the head of the household—lived in a household with the grandchildren of the household head, the status of having children for those persons was judged as uncertain (missing) because it was difficult to determine who was the parent. In addition, only information on children living with their parents in the same household was available in the data.

Regarding the status of having children, we used five types of variables in the analysis. Four variables were based on the ages of the children, and four dummy variables were created: having children aged 0–4 years, having children aged 5–9 years, having children aged 10–14 years, and having children aged 15–19 years. Persons aged 15–19 years were also classified as children because, persons aged ≥ 20 years were classified as adults until 2022 in Japan. For parents with multiple children, the ages of all the children were considered, and we created a dummy variable for each age group based on whether the parents have a child corresponding to that age group or not. In addition, the number of children was also used as another indicator of having children in the analysis, and it was categorized into none, one, two, and three or more.

Because it is rare for older persons to have children aged 0–4 years, persons aged 20–49 years were used in the analysis. In addition, people who had been admitted to a hospital or a nursing home were not included.

### Statistical analysis

The primary analysis was conducted by sex, and another analysis by marital status was also conducted because a previous study demonstrated that the association between having children and smoking status differed depending on marital status [22]. In contrast, an analysis targeting unmarried men was not conducted because single-father households were unavailable in the dataset, and the number of unmarried men with children was small.

We calculated the smoking prevalence and the mean score of the ordinal categorical variable of the number of cigarettes smoked per day by their characteristics and sex, and the analysis was also conducted by marital status. In addition, a modified Poisson regression analysis was conducted to investigate associations of smoking prevalence with child age and the number of children. Robust variance was used to calculate standard errors in the analysis method. Smoking prevalence was used as the outcome variable, and age group, household income, educational attainment, marital status, residential type, employment status, and year were used as the explanatory variables. In addition, dummy variables of having children aged 0–4 years, having children aged 5–9 years, having children aged 10–14 years, and having children aged 15–19 years, and the number of children were used as explanatory variables, while the analyses of the ages of children and the number of children were conducted separately to avoid explanatory variable multicollinearity. In addition, marital status was excluded from the list of explanatory variables in the analysis targeting married persons because all persons were married. Prevalence ratio (PR), 95% confidence intervals (CI), and p-value were calculated for the explanatory variables, and *p* < 0.05 was considered statistically significant. Moreover, an ordinal logistic regression model (proportional odds model) was used to investigate the association between the number of cigarettes smoked per day and having children [23, 24], and the ordinal categorical variable of the number of cigarettes smoked per day was used as the outcome variable. Odds ratio (OR), 95% CI, and p-value were calculated for the explanatory variables in this analysis. Age group and year were used as explanatory variables because smoking status changes depending on those factors in Japan [25, 26], and it is considered that the number and ages of children can also change by those factors. Household income, educational attainment, marital status, residential type, and employment status were used because they are representative indicators of socioeconomic status. Smoking status and the number of children change depending on socioeconomic status in Japan [26–29].

A complete-case analysis was conducted to deal with missing data, and an analysis using multiple imputations by multivariate imputation by chained equations was also conducted as a sensitivity analysis [30]. Predictive mean matching was used as the imputation method, and the number of imputations was set as 50. All statistical analyses were performed using R4.5.0 [31], using lmtest, mice, ordinal, and sandwich as packages. The statistics shown in this study were created by the author using the anonymous data which were provided, and those are statistics published by the Ministry.

## Results

**Fig. 1:**
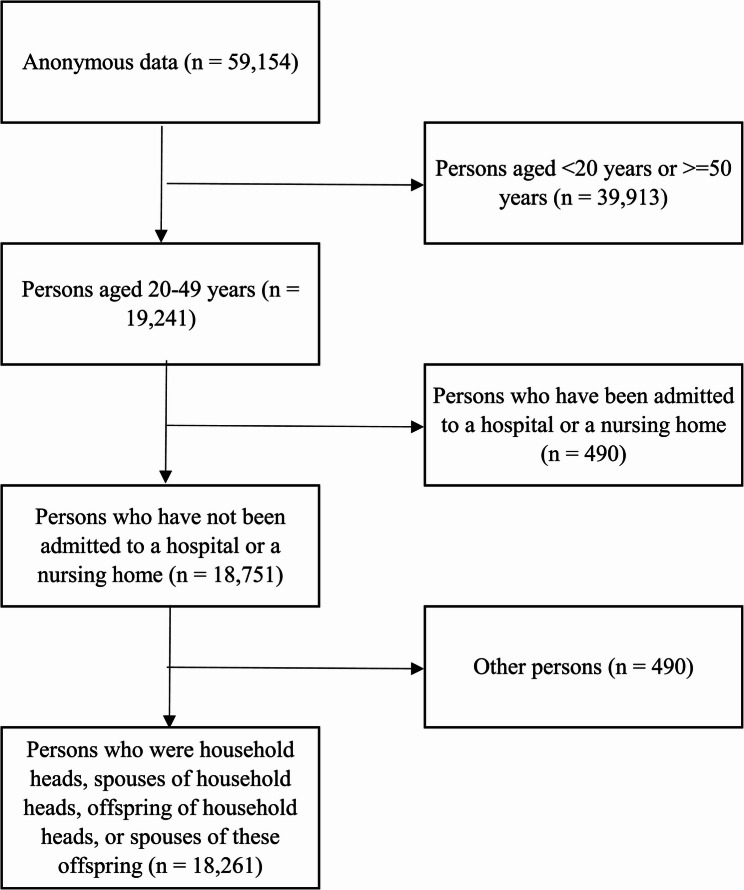
Flowchart of selecting the study population

The flowchart of the participant selection process is presented in Fig. 1. After selecting persons aged 20–49 years, those who had not been admitted to a hospital or a nursing home, and those who were household heads, spouses of household heads, offspring of household heads, or spouses of these offspring, 18,261 persons were used in the analysis.

The number of participants, smoking prevalence, and the mean score of the number of cigarettes smoked per day by the characteristics and sex are presented in Table 1. The number of participants (%) for each category of the number of cigarettes smoked per day among women and men are presented in Supplementary Tables 1 and 2, respectively. Smoking prevalence and the mean score of the number of cigarettes smoked per day in persons who did not have children aged 0–4 years were higher than those in persons who had children aged 0–4 years, among both women and men, and the differences in smoking prevalence and the mean score were larger among women. In contrast, smoking prevalence and the mean score in persons who did not have children aged 5–9 years, 10–14 years, or 15–19 years were lower than those in persons who had such children for each age group of children, among both women and men. In addition, the differences in prevalence and the score between persons with children aged 5–9 years, 10–14 years, or 15–19 years and those without such children were larger among men. In addition, smoking prevalence and the mean score increased with an increase in the number of children among men.

**Table 1:** Number of participants and smoking status by the participant’s characteristics and sex

Characteristics	Women			Men		
	Number of persons	Number of smokers (%)*	Mean score of the number of cigarettes smoked per day*†	Number of persons	Number of smokers (%)*	Mean score of the number of cigarettes smoked per day*†
Total	9,371	1,300 (14.1)	0.23	8,890	3,442 (39.9)	0.75
Age group						
20–24 years	1,072	111 (10.6)	0.15	937	265 (29.2)	0.46
25–29 years	1,096	139 (13.0)	0.19	1,038	388 (38.8)	0.66
30–34 years	1,443	208 (14.7)	0.23	1,345	529 (39.9)	0.71
35–39 years	1,799	267 (15.1)	0.26	1,790	729 (42.2)	0.82
40–44 years	2,079	319 (15.7)	0.27	1,926	796 (42.6)	0.83
45–49 years	1,882	256 (13.8)	0.23	1,854	735 (40.8)	0.81
Marital status						
Married	5,787	755 (13.2)	0.22	5,049	2,042 (41.3)	0.78
Never-married	3,012	371 (12.7)	0.20	3,634	1,275 (36.4)	0.66
Widowed/divorced	572	174 (31.3)	0.54	207	125 (65.1)	1.42
Educational attainment						
Less than high school	271	116 (44.3)	0.81	399	240 (62.0)	1.29
High school	2,998	616 (21.0)	0.34	3,011	1,418 (48.6)	0.93
Professional training college	1,400	194 (14.0)	0.23	1,102	442 (41.3)	0.75
Technical college/Junior college	1,663	115 (7.0)	0.10	259	96 (38.1)	0.72
University	1,843	81 (4.4)	0.07	2,845	793 (28.4)	0.51
Currently enrolled in school	344	18 (5.4)	0.08	411	82 (20.9)	0.32
Missing	852	160 (19.7)	0.33	863	371 (44.9)	0.86
Employment status						
Regular worker	5,188	730 (14.4)	0.23	6,861	2,694 (40.2)	0.75
Non-regular worker	1,043	157 (15.3)	0.25	350	117 (34.8)	0.62
Self-employed worker	452	75 (17.2)	0.29	769	364 (49.5)	1.02
Other workers	88	9 (10.6)	0.20	82	26 (33.3)	0.64
Unemployed person	2,549	321 (12.8)	0.22	751	208 (28.8)	0.51
Missing	51	8 (16.3)	0.22	77	33 (47.8)	0.90
Household income						
Quantile 1 (Lowest)	2,368	481 (20.8)	0.35	2,202	923 (43.5)	0.82
Quantile 2	2,261	347 (15.7)	0.25	2,300	928 (41.6)	0.79
Quantile 3	2,322	266 (11.6)	0.19	2,246	863 (39.3)	0.73
Quantile 4 (Highest)	2,420	206 (8.7)	0.14	2,142	728 (34.9)	0.64
Residential type						
Owning a house	6,452	761 (12.0)	0.19	6,085	2,299 (38.9)	0.72
Rental or employment housing	2,563	461 (18.3)	0.31	2,445	980 (41.0)	0.78
Lodging or others	356	78 (22.7)	0.39	360	163 (47.9)	0.95
Having children aged 0–4 years						
No	7,341	1,086 (15.1)	0.25	6,989	2,710 (40.1)	0.76
Yes	1,985	203 (10.3)	0.16	1,876	725 (39.2)	0.71
Missing	45	11 (25.6)	0.30	25	7 (31.8)	0.59
Having children aged 5–9 years						
No	7,079	971 (14.0)	0.23	6,934	2,616 (38.9)	0.72
Yes	2,247	318 (14.4)	0.24	1,931	819 (43.5)	0.83
Missing	45	11 (25.6)	0.30	25	7 (31.8)	0.59
Having children aged 10–14 years						
No	8,008	1,098 (14.0)	0.23	7,859	3,015 (39.5)	0.74
Yes	1,318	191 (14.8)	0.25	1,006	420 (42.9)	0.83
Missing	45	11 (25.6)	0.30	25	7 (31.8)	0.59
Having children aged 15–19 years						
No	7,795	1,030 (13.5)	0.22	7,880	2,993 (39.1)	0.72
Yes	1,531	259 (17.3)	0.30	985	442 (46.0)	0.93
Missing	45	11 (25.6)	0.30	25	7 (31.8)	0.59
Number of children						
None	4,324	599 (14.2)	0.23	4,827	1,773 (38.1)	0.71
One	1,990	294 (15.0)	0.25	1,501	587 (39.7)	0.75
Two	2,263	297 (13.3)	0.22	1,874	779 (42.5)	0.80
Three or more	749	99 (13.5)	0.22	663	296 (46.0)	0.88
Missing	45	11 (25.6)	0.30	25	7 (31.8)	0.59
Year						
2010	2,588	374 (15.2)	0.26	2,461	992 (43.5)	0.85
2013	2,685	428 (16.1)	0.26	2,507	1,027 (41.4)	0.77
2016	2,374	304 (12.9)	0.21	2,234	832 (37.6)	0.71
2019	1,724	194 (11.4)	0.18	1,688	591 (35.5)	0.63

* The smoking prevalence (%) and the mean score were calculated by excluding persons whose smoking status was missing.

†Scores of 0, 1, 2, and 3 were assigned to the number of cigarettes smoked per day of 0, <=10, 11–20, and >=21 cigarettes, respectively.

Supplementary Table 3 shows the number of participants, smoking prevalence, and the mean score of the number of cigarettes smoked per day by marital status and the participants’ characteristics among women. Smoking prevalence and the mean score of the number of cigarettes smoked per day in women who did not have children aged 0–4 years were higher than those in women who had such children among married women, while the opposite trend was observed among unmarried women. Smoking prevalence and the mean score in women who had children aged 5–9 years, aged 10–14 years, or 15–19 years were higher than those in women who did not have such children among unmarried women for each age group of children. In addition, regarding the number of children, smoking prevalence and the mean score in women with no children were the highest and lowest among married and unmarried women, respectively.

Supplementary Table 4 shows the number of participants, smoking prevalence, and the mean score of the number of cigarettes smoked per day by the characteristics among married men. The pattern for the association between having children and smoking status was similar to that in men overall.

Table 2 shows the results of the regression analyses investigating associations between the ages of children and smoking prevalence by sex and marital status. Having children aged 0–4 years was associated with lower smoking prevalence in women overall, and the adjusted PR was 0.62 (95% CI: 0.52, 0.74). Statistically significant associations with lower smoking prevalence were also observed among married women and men, with adjusted PRs of 0.61 (95% CI: 0.51, 0.74) and 0.91 (95% CI: 0.84, 0.99), respectively. In addition, having children aged 5–9 years and having children aged 15–19 years were significantly associated with higher smoking prevalence in married men, and the adjusted PRs were 1.10 (95% CI: 1.02, 1.18) and 1.12 (95% CI: 1.02, 1.24), respectively. Moreover, having children aged 0–4 years was not significantly associated with smoking prevalence in unmarried women, with a PR of 0.88 (95% CI: 0.59, 1.30), which was higher than that among married women.

**Table 2:** Results of the regression analyses investigating associations between the ages of children and smoking prevalence

Marital status and the ages of children	Women		Men	
	Adjusted PR (95% CI)*	*p*-value	Adjusted PR (95% CI)*	*p*-value
All persons				
Having children aged 0–4 years (Reference: No)	0.62 (0.52, 0.74)	< 0.001	0.92 (0.85, 1.00)	0.054
Having children aged 5–9 years (Reference: No)	0.99 (0.86, 1.13)	0.858	1.08 (1.01, 1.16)	0.034
Having children aged 10–14 years (Reference: No)	0.85 (0.72, 1.00)	0.054	0.98 (0.89, 1.07)	0.587
Having children aged 15–19 years (Reference: No)	1.10 (0.94, 1.28)	0.246	1.10 (1.00, 1.20)	0.054
Married persons				
Having children aged 0–4 years (Reference: No)	0.61 (0.51, 0.74)	< 0.001	0.91 (0.84, 0.99)	0.029
Having children aged 5–9 years (Reference: No)	0.96 (0.82, 1.12)	0.609	1.10 (1.02, 1.18)	0.012
Having children aged 10–14 years (Reference: No)	0.85 (0.71, 1.03)	0.09	0.99 (0.91, 1.09)	0.914
Having children aged 15–19 years (Reference: No)	1.10 (0.91, 1.32)	0.334	1.12 (1.02, 1.24)	0.02
Unmarried persons				
Having children aged 0–4 years (Reference: No)	0.88 (0.59, 1.30)	0.509	-	-
Having children aged 5–9 years (Reference: No)	1.01 (0.76, 1.35)	0.927	-	-
Having children aged 10–14 years (Reference: No)	0.75 (0.54, 1.05)	0.092	-	-
Having children aged 15–19 years (Reference: No)	1.12 (0.84, 1.49)	0.453	-	-

*PR* prevalence ratio, *CI* confidence interval

*Age group, household income, educational attainment, marital status, residential type, employment status, and year were adjusted as the explanatory variables, while marital status was not used in the analysis of married persons.

Supplementary Table 5 shows the results of the regression analyses investigating associations between the ages of children and smoking prevalence by sex and marital status using multiple imputation. These findings were similar to those obtained in the complete-case analysis, while having children aged 15–19 years was significantly associated with a higher smoking prevalence in men overall, in contrast to the complete-case analysis.

Table 3 shows the results of the regression analyses investigating associations between the number of children and smoking prevalence by sex and marital status. The number of children was inversely proportional to the PR for smoking in women overall, and having 2 children and having ≥ 3 children were significantly associated with lower smoking prevalence, with adjusted PRs of 0.82 (95% CI:0.69, 0.97) and 0.76 (95% CI:0.60, 0.96), respectively. A similar association was observed among married women, while significant associations were not found among unmarried women. In contrast, the number of children was not significantly associated with smoking prevalence in men overall, while having ≥ 3 children was significantly associated with a higher smoking prevalence in married men, with a PR of 1.13 (95% CI: 1.00, 1.27).

**Table 3:** Results of the regression analyses investigating associations between the number of children and smoking prevalence

Marital status and the number of children	Women		Men	
	Adjusted PR (95% CI)*	*p*-value	Adjusted PR (95% CI)*	*p*-value
All persons				
None	Reference		Reference	
One	0.92 (0.78, 1.08)	0.307	0.99 (0.90, 1.10)	0.906
Two	0.82 (0.69, 0.97)	0.021	1.05 (0.95, 1.15)	0.367
Three or more	0.76 (0.60, 0.96)	0.020	1.11 (0.98, 1.25)	0.089
Married persons				
None	Reference		Reference	
One	0.88 (0.72, 1.06)	0.180	1.00 (0.90, 1.12)	0.926
Two	0.80 (0.66, 0.96)	0.020	1.07 (0.97, 1.18)	0.200
Three or more	0.71 (0.55, 0.92)	0.009	1.13 (1.00, 1.27)	0.047
Unmarried persons				
None	Reference		-	-
One	1.05 (0.76, 1.44)	0.773	-	-
Two	0.84 (0.59, 1.21)	0.350	-	-
Three or more	1.06 (0.60, 1.87)	0.850	-	-

*PR* prevalence ratio, *CI* confidence interval

*Age group, household income, educational attainment, marital status, residential type, employment status, and year were adjusted as the explanatory variables, while marital status was not used in the analysis of married persons.

Supplementary Table 6 shows the results of the regression analyses investigating associations between the number of children and smoking prevalence by sex and marital status using multiple imputation. A similar significant association was observed as in the complete-case analysis.

Table 4 shows the results of the regression analyses investigating associations between the ages of children and the number of cigarettes smoked per day by sex and marital status. The results were similar to those of smoking prevalence in women, and having children aged 0–4 years was significantly associated with a lower number of cigarettes smoked per day in women overall and in married women, with the adjusted ORs of 0.55 (95% CI: 0.45, 0.68) and 0.54 (95% CI: 0.43, 0.68), respectively. Similar to the results of smoking prevalence, having children aged 5–9 years and having children aged 15–19 years were significantly associated with a higher number of cigarettes smoked per day in men. In contrast, having children aged 0–4 years was significantly associated with a lower number of cigarettes smoked per day in men overall and in married men, with the adjusted ORs of 0.83 (95% CI: 0.73, 0.96) and 0.82 (95% CI: 0.72, 0.95), respectively.

**Table 4:** Results of the regression analyses investigating associations between the ages of children and smoking intensity

Marital status and the ages of children	Women		Men	
	Adjusted OR (95% CI)*	*p*-value	Adjusted OR (95% CI)*	*p*-value
All persons				
Having children aged 0–4 years (Reference: No)	0.55 (0.45, 0.68)	< 0.001	0.83 (0.73, 0.96)	0.009
Having children aged 5–9 years (Reference: No)	0.95 (0.80, 1.12)	0.533	1.14 (1.01, 1.29)	0.039
Having children aged 10–14 years (Reference: No)	0.82 (0.67, 1.01)	0.056	0.92 (0.79, 1.08)	0.329
Having children aged 15–19 years (Reference: No)	1.14 (0.93, 1.39)	0.207	1.26 (1.06, 1.49)	0.007
Married persons				
Having children aged 0–4 years (Reference: No)	0.54 (0.43, 0.68)	< 0.001	0.82 (0.72, 0.95)	0.006
Having children aged 5–9 years (Reference: No)	0.93 (0.77, 1.12)	0.441	1.17 (1.02, 1.33)	0.02
Having children aged 10–14 years (Reference: No)	0.82 (0.65, 1.03)	0.086	0.95 (0.81, 1.12)	0.541
Having children aged 15–19 years (Reference: No)	1.13 (0.90, 1.42)	0.278	1.31 (1.10, 1.56)	0.002
Unmarried persons				
Having children aged 0–4 years (Reference: No)	0.86 (0.47, 1.60)	0.64	-	-
Having children aged 5–9 years (Reference: No)	0.89 (0.57, 1.41)	0.631	-	-
Having children aged 10–14 years (Reference: No)	0.66 (0.40, 1.09)	0.105	-	-
Having children aged 15–19 years (Reference: No)	1.16 (0.74, 1.81)	0.518	-	-

*OR* odds ratio, *CI* confidence interval

*Age group, household income, educational attainment, marital status, residential type, employment status, and year were adjusted as the explanatory variables, while marital status was not used in the analysis of married persons.

Supplementary Table 7 shows the results of the regression analyses investigating associations between the ages of children and the number of cigarettes smoked per day by sex and marital status using multiple imputation. These findings were similar to those obtained in the complete-case analysis.

Table 5 shows the results of the regression analyses investigating associations between the number of children and the number of cigarettes smoked per day by sex and marital status. Similar to the results of smoking prevalence, having 2 children and having ≥ 3 children were significantly associated with a lower number of cigarettes smoked per day in women overall and in married women, while significant associations were not observed in men and in unmarried women.

**Table 5:** Results of the regression analyses investigating associations between the number of children and smoking intensity

Marital status and the number of children	Women		Men	
	Adjusted OR (95% CI)*	*p*-value	Adjusted OR (95% CI)*	*p*-value
All persons				
None	Reference		Reference	
One	0.90 (0.73, 1.11)	0.320	0.96 (0.81, 1.13)	0.607
Two	0.75 (0.61, 0.93)	0.009	1.05 (0.89, 1.24)	0.560
Three or more	0.68 (0.51, 0.91)	0.009	1.14 (0.93, 1.41)	0.202
Married persons				
None	Reference		Reference	
One	0.84 (0.66, 1.06)	0.134	0.98 (0.83, 1.17)	0.845
Two	0.74 (0.58, 0.93)	0.011	1.08 (0.91, 1.28)	0.356
Three or more	0.63 (0.46, 0.86)	0.004	1.17 (0.95, 1.45)	0.137
Unmarried persons				
None	Reference		-	-
One	1.13 (0.70, 1.83)	0.613	-	-
Two	0.68 (0.39, 1.18)	0.171	-	-
Three or more	1.09 (0.45, 2.60)	0.851	-	-

*OR* odds ratio, *CI* confidence interval

*Age group, household income, educational attainment, marital status, residential type, employment status, and year were adjusted as the explanatory variables, while marital status was not used in the analysis of married persons.

Supplementary Table 8 shows the results of the regression analyses investigating associations between the number of children and the number of cigarettes smoked per day by sex and marital status using multiple imputation. These findings were similar to those obtained in the complete-case analysis.

## Discussion

Smoking prevalence and intensity differed depending on having children, and the association changed depending on parental sex and marital status, as well as the ages and numbers of children. In this section, we discuss possible reasons for the results and limitations of the study.

Having children aged 0–4 years was associated with lower smoking prevalence or fewer cigarettes smoked per day among women and men overall, and the association was evident among married persons. Similar associations among married women were observed in studies in Korea and the United States [13, 22]. It is known that smoking cessation occurs during pregnancy and after having an infant among parents [12, 13]. In addition, the association between lower smoking prevalence and having children was stronger among women, which was consistent with the findings of a study conducted in the United Kingdom [14]. In contrast, another study performed in the United Kingdom demonstrated that smoking behavior did not change among the majority of fathers of new infants [32], and a study conducted in Sweden also showed that parenthood was not associated with a lower smoking prevalence among men and women [15].

In addition, the significant associations of having children aged 0–4 years with smoking prevalence and intensity were shown only among married women, not among unmarried women. The lower number of unmarried mothers is a possible reason because the PR of smoking prevalence and the OR of the number of cigarettes smoked per day for having children aged 0–4 years were < 1 also among unmarried women. However, the PR and OR increased compared to those among married women, and it was suggested that having children aged 0–4 years was associated with lower smoking prevalence and fewer cigarettes smoked per day more among married women than among unmarried women. A similar result was obtained in a previous study performed in the United States [22], in which it was pointed out that single mothers with lower socioeconomic status tended to face more stressors, triggering smoking [22]. In addition, socioeconomic status is known to be associated with smoking cessation and relapses [18, 33, 34]. Moreover, partner support was shown to be an important determinant of smoking cessation [35], and it is considered that single women have less support than married ones [22].

In addition, having children aged 5–9 years, 10–14 years, or 15–19 years was not significantly associated with smoking status in women. In contrast, there was a significantly positive association of having children aged 5–9 years and having children aged 15–19 years with smoking prevalence and the number of cigarettes in men overall. A previous study conducted in Germany demonstrated that women’s smoking probability drops until the first child turns 18 [36], while no such association was observed in this study. In addition, a previous study conducted in the United States showed that being a single woman having children aged 5–17 years was significantly associated with a higher smoking prevalence compared to being one without a child [22]. According to another study in the United States, adults were more likely to smoke when they were living with children [37]. A study performed in Japan showed that the smoking prevalence increases from gestation to 18 months postpartum among women [12], and another study conducted in Korea showed that a relapse of smoking occurred as children grew among former smokers [13]. It is possible that smoking cessation occurs only during a short postpartum period in men and women in Japan because it is recommended mainly during gestation and while a child is in its infancy. In Japan, health checkups for young children aged 0–4 years, particularly for infants, are conducted periodically by local governments [38], and parents have opportunities to receive guidance for smoking cessation. In contrast, such opportunities become fewer as a child grows, which might have led to the results in our study. In contrast, the reason for the significant positive associations of having children aged 5–9 years and having children aged 15–19 years with smoking prevalence and the number of cigarettes in men is uncertain.

Our study also demonstrated that having a higher number of children was associated with lower smoking prevalence and intensity among women, and this association was not observed in unmarried women. A similar association was shown in a study conducted in the United Kingdom [14], and this study also demonstrated a less apparent association among men, which was consistent with the findings of our study. A study conducted in China also indicated that the number of children was negatively associated with smoking prevalence among family members [17]. In contrast, having three or more children was positively associated with the prevalence of smoking among married men in our study. A study performed in Vietnam showed a positive association between smoking and the number of children [16], which was explained by the increased stress levels among parents of multiple children. It is pointed out that an experience of pregnancy is one reason why women are more responsible for being a parent than men, according to a study conducted in the United Kingdom [14], and it can also hold true in Japan. In addition, mothers tend to be more involved in raising children than fathers in Japan [39], and mothers typically spend more time with the children compared with fathers [40]. Therefore, it is possible that mothers tend to recognize the need for smoking cessation more than fathers. Similarly, a report indicated that mothers accompanied young children during health checkups more than fathers in Japan [41], and the difference in the number of opportunities for participating in health checkups for young children between parents can lead to differences in smoking cessation.

Having children was associated with lower smoking and intensity, while the association changed depending on multiple factors, including children’s ages. Because children aged ≥ 5 years also suffer the negative effects of their parents’ smoking [7, 42, 43], smoking cessation efforts are also required among parents having children aged ≥ 5 years. In addition, the associations of having children aged 0–4 years with lower smoking prevalence and intensity were less apparent among men and were not apparent in unmarried women. Smoking prevalence is higher among men than among women in Japan, and paternal smoking affects child health. It is important to promote smoking cessation among fathers and to investigate the reasons for the positive association of smoking with having children aged ≥ 5 years and having a higher number of children among men.

Nevertheless, this study has some limitations that should be acknowledged. Households with ≥ 8 members were excluded from the anonymous data used in this study by the Ministry of Health, Labour and Welfare. In addition, an analysis of the association between having children and smoking status among unmarried men could not be conducted because single-father households were excluded from the anonymized dataset. Additionally, only the 5-year age group was available as information on age in the data. In the future, it will be relevant to examine the association using the individual-level data of the Comprehensive Survey of Living Conditions, which are different from the anonymous data, or other survey data to circumvent the limitations of the data employed in this study. Additionally, the presence of pregnant women or breastfeeding women in the household has not been investigated in the survey, while these factors can affect smoking status. Moreover, we used the family relationship with the head of the household to identify the status of having children for the children of the household head and their spouses, and there were some cases in which it was difficult to identify the status of having children exactly from the data. Furthermore, this study used repeated cross-sectional data, and it is difficult to establish a causal relationship between smoking and having children. It is important to investigate the association using longitudinal data in future studies. In contrast, the main strength of the study is that we used the nationally representative survey data collected by the government, which ensures the reliability and generalizability of our findings.

## Conclusions

Having children aged 0–4 years was associated with lower smoking prevalence or fewer cigarettes smoked per day both in women and men overall, but no such significant association was observed in unmarried women. In addition, having children aged 5–9 years, 10–14 years, or 15–19 years was not significantly associated with a lower smoking prevalence in men and women, and having children aged 5–9 years and having children aged 15–19 years were significantly associated with a higher number of cigarettes smoked per day in men overall. Moreover, there was a significant inverse association between the number of children and smoking prevalence in women overall, while such association was not observed in men. The results suggested the need for smoking cessation campaigns against men and unmarried women with young children because they might have fewer opportunities to be informed about the importance of the same than married women at present. In addition, the results also suggested the need for increasing public opportunities for checking smoking status against parents with school-aged children and adolescents in Japan because those opportunities may become fewer as the children grow.

## Supplementary Information

Supplementary Material 1.

## Data Availability

The data that support the findings of this study are available from the Ministry of Health, Labour and Welfare in Japan.

## Abbreviations

OR: Odds ratio
PR: Prevalence ratio
CI: Confidence interval
