# Supplementary material for: An analysis of associations of having children with smoking prevalence and intensity using nationally representative survey data in Japan
Source: BMC Public Health. 2026 Feb 27;26:1099. doi: 10.1186/s12889-026-26817-3 (PMC13049891; doi:10.1186/s12889-026-26817-3)
Supplement: Supplementary file 1 — Supplementary Material 1. [file 12889_2026_26817_MOESM1_ESM.pdf]

Supplementary Table 1. Number of participants (%) for each category of the number of cigarettes smoked per day among women

Characteristics	Number of cigarettes smoked per day					
	0 cigarettes	<=10 cigarettes	11–20 cigarettes	21 or more cigarettes	Smokers without information on the number of cigarettes	Persons without information on smoking status
Total	7,890 (84.2)	572 (6.1)	603 (6.4)	116 (1.2)	9 (0.1)	181 (1.9)
Age group						
20–24 years	940 (87.7)	66 (6.2)	40 (3.7)	5 (0.5)	0 (0.0)	21 (2.0)
25–29 years	932 (85.0)	80 (7.3)	51 (4.7)	6 (0.5)	2 (0.2)	25 (2.3)
30–34 years	1,208 (83.7)	104 (7.2)	92 (6.4)	11 (0.8)	1 (0.1)	27 (1.9)
35–39 years	1,504 (83.6)	101 (5.6)	138 (7.7)	28 (1.6)	0 (0.0)	28 (1.6)
40–44 years	1,713 (82.4)	115 (5.5)	161 (7.7)	40 (1.9)	3 (0.1)	47 (2.3)
45–49 years	1,593 (84.6)	106 (5.6)	121 (6.4)	26 (1.4)	3 (0.2)	33 (1.8)
Marital status						
Married	4,954 (85.6)	316 (5.5)	373 (6.4)	60 (1.0)	6 (0.1)	78 (1.3)
Never-married	2,554 (84.8)	184 (6.1)	153 (5.1)	31 (1.0)	3 (0.1)	87 (2.9)
Widowed/divorced	382 (66.8)	72 (12.6)	77 (13.5)	25 (4.4)	0 (0.0)	16 (2.8)
Educational attainment						
Less than high school	146 (53.9)	37 (13.7)	61 (22.5)	18 (6.6)	0 (0.0)	9 (3.3)
High school	2,316 (77.3)	259 (8.6)	309 (10.3)	43 (1.4)	5 (0.2)	66 (2.2)
Professional training college	1,188 (84.9)	83 (5.9)	91 (6.5)	19 (1.4)	1 (0.1)	18 (1.3)
Technical college/Junior college	1,529 (91.9)	64 (3.8)	45 (2.7)	6 (0.4)	0 (0.0)	19 (1.1)
University	1,744 (94.6)	45 (2.4)	30 (1.6)	6 (0.3)	0 (0.0)	18 (1.0)
Currently enrolled in school	315 (91.6)	10 (2.9)	7 (2.0)	1 (0.3)	0 (0.0)	11 (3.2)

Missing	652 (76.5)	74 (8.7)	60 (7.0)	23 (2.7)	3 (0.4)	40 (4.7)
Employment status						
Regular worker	4,350 (83.8)	333 (6.4)	337 (6.5)	55 (1.1)	5 (0.1)	108 (2.1)
Non-regular worker	868 (83.2)	67 (6.4)	80 (7.7)	10 (1.0)	0 (0.0)	18 (1.7)
Self-employed worker	362 (80.1)	31 (6.9)	33 (7.3)	10 (2.2)	1 (0.2)	15 (3.3)
Other workers	76 (86.4)	3 (3.4)	4 (4.5)	2 (2.3)	0 (0.0)	3 (3.4)
Unemployed person	2,193 (86.0)	132 (5.2)	148 (5.8)	38 (1.5)	3 (0.1)	35 (1.4)
Missing	41 (80.4)	6 (11.8)	1 (2.0)	1 (2.0)	0 (0.0)	2 (3.9)
Household income						
Quantile 1 (Lowest)	1,826 (77.1)	190 (8.0)	237 (10.0)	50 (2.1)	4 (0.2)	61 (2.6)
Quantile 2	1,868 (82.6)	157 (6.9)	156 (6.9)	31 (1.4)	3 (0.1)	46 (2.0)
Quantile 3	2,022 (87.1)	123 (5.3)	121 (5.2)	20 (0.9)	2 (0.1)	34 (1.5)
Quantile 4 (Highest)	2,174 (89.8)	102 (4.2)	89 (3.7)	15 (0.6)	0 (0.0)	40 (1.7)
Residential type						
Owning a house	5,559 (86.2)	359 (5.6)	337 (5.2)	59 (0.9)	6 (0.1)	132 (2.0)
Rental or employment housing	2,065 (80.6)	183 (7.1)	230 (9.0)	47 (1.8)	1 (0.0)	37 (1.4)
Lodging or others	266 (74.7)	30 (8.4)	36 (10.1)	10 (2.8)	2 (0.6)	12 (3.4)
Having children aged 0–4 years						
No	6,093 (83.0)	464 (6.3)	516 (7.0)	99 (1.3)	7 (0.1)	162 (2.2)
Yes	1,765 (88.9)	99 (5.0)	85 (4.3)	17 (0.9)	2 (0.1)	17 (0.9)
Missing	32 (71.1)	9 (20.0)	2 (4.4)	0 (0.0)	0 (0.0)	2 (4.4)
Having children aged 5–9 years						
No	5,970 (84.3)	424 (6.0)	448 (6.3)	91 (1.3)	8 (0.1)	138 (1.9)
Yes	1,888 (84.0)	139 (6.2)	153 (6.8)	25 (1.1)	1 (0.0)	41 (1.8)
Missing	32 (71.1)	9 (20.0)	2 (4.4)	0 (0.0)	0 (0.0)	2 (4.4)
Having children aged 10–14 years						
No	6,757 (84.4)	490 (6.1)	503 (6.3)	97 (1.2)	8 (0.1)	153 (1.9)
Yes	1,101 (83.5)	73 (5.5)	98 (7.4)	19 (1.4)	1 (0.1)	26 (2.0)

Missing	32 (71.1)	9 (20.0)	2 (4.4)	0 (0.0)	0 (0.0)	2 (4.4)
Having children aged 15–19 years						
No	6,617 (84.9)	470 (6.0)	465 (6.0)	87 (1.1)	8 (0.1)	148 (1.9)
Yes	1,241 (81.1)	93 (6.1)	136 (8.9)	29 (1.9)	1 (0.1)	31 (2.0)
Missing	32 (71.1)	9 (20.0)	2 (4.4)	0 (0.0)	0 (0.0)	2 (4.4)
Number of children						
None	3,626 (83.9)	267 (6.2)	271 (6.3)	55 (1.3)	6 (0.1)	99 (2.3)
One	1,667 (83.8)	124 (6.2)	139 (7.0)	30 (1.5)	1 (0.1)	29 (1.5)
Two	1,929 (85.2)	130 (5.7)	143 (6.3)	23 (1.0)	1 (0.0)	37 (1.6)
Three or more	636 (84.9)	42 (5.6)	48 (6.4)	8 (1.1)	1 (0.1)	14 (1.9)
Missing	32 (71.1)	9 (20.0)	2 (4.4)	0 (0.0)	0 (0.0)	2 (4.4)
Year						
2010	2,083 (80.5)	157 (6.1)	173 (6.7)	42 (1.6)	2 (0.1)	131 (5.1)
2013	2,238 (83.4)	191 (7.1)	199 (7.4)	37 (1.4)	1 (0.0)	19 (0.7)
2016	2,055 (86.6)	134 (5.6)	143 (6.0)	23 (1.0)	4 (0.2)	15 (0.6)
2019	1,514 (87.8)	90 (5.2)	88 (5.1)	14 (0.8)	2 (0.1)	16 (0.9)

Supplementary Table 2. Number of participants (%) for each category of the number of cigarettes smoked per day among men

Characteristics	Number of cigarettes smoked per day					
	0 cigarettes	<=10 cigarettes	11–20 cigarettes	21 or more cigarettes	Smokers without information on the number of cigarettes	Persons without information on smoking status
Total	5,194 (58.4)	986 (11.1)	1,855 (20.9)	578 (6.5)	23 (0.3)	254 (2.9)
Age group						
20–24 years	643 (68.6)	129 (13.8)	118 (12.6)	16 (1.7)	2 (0.2)	29 (3.1)
25–29 years	613 (59.1)	147 (14.2)	206 (19.8)	33 (3.2)	2 (0.2)	37 (3.6)
30–34 years	797 (59.3)	176 (13.1)	279 (20.7)	70 (5.2)	4 (0.3)	19 (1.4)
35–39 years	1,000 (55.9)	186 (10.4)	399 (22.3)	143 (8.0)	1 (0.1)	61 (3.4)
40–44 years	1,073 (55.7)	192 (10.0)	436 (22.6)	163 (8.5)	5 (0.3)	57 (3.0)
45–49 years	1,068 (57.6)	156 (8.4)	417 (22.5)	153 (8.3)	9 (0.5)	51 (2.8)
Marital status						
Married	2,901 (57.5)	547 (10.8)	1,127 (22.3)	351 (7.0)	17 (0.3)	106 (2.1)
Never-married	2,226 (61.3)	422 (11.6)	660 (18.2)	187 (5.1)	6 (0.2)	133 (3.7)
Widowed/divorced	67 (32.4)	17 (8.2)	68 (32.9)	40 (19.3)	0 (0.0)	15 (7.2)
Educational attainment						
Less than high school	147 (36.8)	46 (11.5)	127 (31.8)	66 (16.5)	1 (0.3)	12 (3.0)
High school	1,497 (49.7)	369 (12.3)	794 (26.4)	250 (8.3)	5 (0.2)	96 (3.2)
Professional training college	629 (57.1)	148 (13.4)	224 (20.3)	67 (6.1)	3 (0.3)	31 (2.8)
Technical college/Junior college	156 (60.2)	23 (8.9)	55 (21.2)	16 (6.2)	2 (0.8)	7 (2.7)

University	2,000 (70.3)	262 (9.2)	426 (15.0)	103 (3.6)	2 (0.1)	52 (1.8)
Currently enrolled in school	310 (75.4)	44 (10.7)	31 (7.5)	6 (1.5)	1 (0.2)	19 (4.6)
Missing	455 (52.7)	94 (10.9)	198 (22.9)	70 (8.1)	9 (1.0)	37 (4.3)
Employment status						
Regular worker	4,002 (58.3)	788 (11.5)	1,461 (21.3)	429 (6.3)	16 (0.2)	165 (2.4)
Non-regular worker	219 (62.6)	38 (10.9)	60 (17.1)	16 (4.6)	3 (0.9)	14 (4.0)
Self-employed worker	371 (48.2)	63 (8.2)	214 (27.8)	86 (11.2)	1 (0.1)	34 (4.4)
Other workers	52 (63.4)	8 (9.8)	12 (14.6)	6 (7.3)	0 (0.0)	4 (4.9)
Unemployed person	514 (68.4)	81 (10.8)	89 (11.9)	36 (4.8)	2 (0.3)	29 (3.9)
Missing	36 (46.8)	8 (10.4)	19 (24.7)	5 (6.5)	1 (1.3)	8 (10.4)
Household income						
Quantile 1 (Lowest)	1,197 (54.4)	265 (12.0)	475 (21.6)	174 (7.9)	9 (0.4)	82 (3.7)
Quantile 2	1,304 (56.7)	249 (10.8)	517 (22.5)	158 (6.9)	4 (0.2)	68 (3.0)
Quantile 3	1,333 (59.3)	254 (11.3)	464 (20.7)	137 (6.1)	8 (0.4)	50 (2.2)
Quantile 4 (Highest)	1,360 (63.5)	218 (10.2)	399 (18.6)	109 (5.1)	2 (0.1)	54 (2.5)
Residential type						
Owning a house	3,604 (59.2)	673 (11.1)	1,245 (20.5)	364 (6.0)	17 (0.3)	182 (3.0)
Rental or employment housing	1,413 (57.8)	275 (11.2)	520 (21.3)	179 (7.3)	6 (0.2)	52 (2.1)
Lodging or others	177 (49.2)	38 (10.6)	90 (25.0)	35 (9.7)	0 (0.0)	20 (5.6)
Having children aged 0–4 years						
No	4,056 (58.0)	751 (10.7)	1,459 (20.9)	480 (6.9)	20 (0.3)	223 (3.2)
Yes	1,123 (59.9)	234 (12.5)	390 (20.8)	98 (5.2)	3 (0.2)	28 (1.5)
Missing	15 (60.0)	1 (4.0)	6 (24.0)	0 (0.0)	0 (0.0)	3 (12.0)

Having children aged 5–9 years						
No	4,117 (59.4)	770 (11.1)	1,393 (20.1)	438 (6.3)	15 (0.2)	201 (2.9)
Yes	1,062 (55.0)	215 (11.1)	456 (23.6)	140 (7.3)	8 (0.4)	50 (2.6)
Missing	15 (60.0)	1 (4.0)	6 (24.0)	0 (0.0)	0 (0.0)	3 (12.0)
Having children aged 10–14 years						
No	4,621 (58.8)	879 (11.2)	1,621 (20.6)	496 (6.3)	19 (0.2)	223 (2.8)
Yes	558 (55.5)	106 (10.5)	228 (22.7)	82 (8.2)	4 (0.4)	28 (2.8)
Missing	15 (60.0)	1 (4.0)	6 (24.0)	0 (0.0)	0 (0.0)	3 (12.0)
Having children aged 15–19 years						
No	4,661 (59.1)	899 (11.4)	1,604 (20.4)	474 (6.0)	16 (0.2)	226 (2.9)
Yes	518 (52.6)	86 (8.7)	245 (24.9)	104 (10.6)	7 (0.7)	25 (2.5)
Missing	15 (60.0)	1 (4.0)	6 (24.0)	0 (0.0)	0 (0.0)	3 (12.0)
Number of children						
None	2,885 (59.8)	534 (11.1)	942 (19.5)	291 (6.0)	6 (0.1)	169 (3.5)
One	891 (59.4)	173 (11.5)	296 (19.7)	111 (7.4)	7 (0.5)	23 (1.5)
Two	1,055 (56.3)	204 (10.9)	435 (23.2)	130 (6.9)	10 (0.5)	40 (2.1)
Three or more	348 (52.5)	74 (11.2)	176 (26.5)	46 (6.9)	0 (0.0)	19 (2.9)
Missing	15 (60.0)	1 (4.0)	6 (24.0)	0 (0.0)	0 (0.0)	3 (12.0)
Year						
2010	1,288 (52.3)	246 (10.0)	537 (21.8)	203 (8.2)	6 (0.2)	181 (7.4)
2013	1,454 (58.0)	303 (12.1)	550 (21.9)	167 (6.7)	7 (0.3)	26 (1.0)
2016	1,378 (61.7)	229 (10.3)	463 (20.7)	134 (6.0)	6 (0.3)	24 (1.1)
2019	1,074 (63.6)	208 (12.3)	305 (18.1)	74 (4.4)	4 (0.2)	23 (1.4)

Supplementary Table 3. Number of participants, smoking prevalence, and the mean score of the number of cigarettes smoked per day by marital status and the participants' characteristics among women

Characteristics	Married women			Unmarried women		
	Number of persons	Number of smokers (%)*	Mean score of the number of cigarettes smoked per day *†	Number of persons	Number of smokers (%)*	Mean score of the number of cigarettes smoked per day *†
Total	5,787	755 (13.2)	0.22	3,584	545 (15.7)	0.25
Age group						
20–24 years	92	21 (22.8)	0.34	980	90 (9.4)	0.14
25–29 years	389	46 (11.9)	0.17	707	93 (13.6)	0.19
30–34 years	914	112 (12.4)	0.19	529	96 (18.8)	0.29
35–39 years	1,321	169 (13.0)	0.23	478	98 (21.0)	0.36
40–44 years	1,601	232 (14.7)	0.25	478	87 (19.0)	0.36
45–49 years	1,470	175 (12.1)	0.20	412	81 (20.1)	0.35
Marital status						
Married	5,787	755 (13.2)	0.22	-	-	-
Never-married	-	-	-	3,012	371 (12.7)	0.20
Widowed/divorced	-	-		572	174 (31.3)	0.54
Educational attainment						
Less than high school	143	62 (44.6)	0.78	128	54 (43.9)	0.85
High school	1,977	370 (19.0)	0.31	1,021	246 (24.9)	0.41
Professional training college	876	113 (13.0)	0.23	524	81 (15.8)	0.25
Technical college/Junior college	1,184	70 (6.0)	0.09	479	45 (9.5)	0.13
University	1,082	43 (4.0)	0.06	761	38 (5.1)	0.08
Currently enrolled in school	17	2 (11.8)	0.18	327	16 (5.1)	0.08

Missing	508	95 (19.3)	0.32	344	65 (20.3)	0.33
Employment status						
Regular worker	2,738	365 (13.5)	0.22	2,450	365 (15.3)	0.24
Non-regular worker	667	92 (14.0)	0.23	376	65 (17.8)	0.29
Self-employed worker	346	51 (15.1)	0.23	106	24 (24.2)	0.48
Other workers	50	5 (10.4)	0.21	38	4 (10.8)	0.19
Unemployed person	1,970	241 (12.4)	0.21	579	80 (14.2)	0.24
Missing	16	1 (6.7)	0.07	35	7 (20.6)	0.29
Household income						
Quantile 1 (Lowest)	1,068	193 (18.5)	0.30	1,300	288 (22.8)	0.39
Quantile 2	1,553	241 (15.7)	0.26	708	106 (15.6)	0.23
Quantile 3	1,658	189 (11.5)	0.19	664	77 (11.9)	0.18
Quantile 4 (Highest)	1,508	132 (8.8)	0.14	912	74 (8.3)	0.12
Residential type						
Owning a house	3,954	456 (11.7)	0.19	2,498	305 (12.6)	0.19
Rental or employment housing	1,604	254 (15.9)	0.27	959	207 (22.2)	0.38
Lodging or others	229	45 (20.5)	0.33	127	33 (26.6)	0.48
Having children aged 0–4 years						
No	3,871	572 (15.0)	0.25	3,470	514 (15.2)	0.25
Yes	1,916	183 (9.6)	0.15	69	20 (29.9)	0.48
Missing				45	11 (25.6)	0.30
Having children aged 5–9 years						
No	3,695	485 (13.3)	0.22	3,384	486 (14.8)	0.24
Yes	2,092	270 (13.1)	0.22	155	48 (31.6)	0.50
Missing				45	11 (25.6)	0.30
Having children aged 10–14 years						
No	4,597	596 (13.1)	0.21	3,411	502 (15.1)	0.25
Yes	1,190	159 (13.6)	0.23	128	32 (26.0)	0.45

Missing				45	11 (25.6)	0.30
Having children aged 15–19 years						
No	4,457	559 (12.7)	0.21	3,338	471 (14.5)	0.23
Yes	1,330	196 (15.0)	0.26	201	63 (32.6)	0.59
Missing				45	11 (25.6)	0.30
Number of children						
None	1,220	195 (16.1)	0.27	3,104	404 (13.4)	0.22
One	1,754	219 (12.7)	0.21	236	75 (32.6)	0.57
Two	2,096	253 (12.2)	0.20	167	44 (27.5)	0.43
Three or more	717	88 (12.5)	0.20	32	11 (35.5)	0.61
Missing				45	11 (25.6)	0.30
Year						
2010	1,675	222 (13.7)	0.24	913	152 (18.1)	0.29
2013	1,631	236 (14.5)	0.24	1,054	192 (18.5)	0.30
2016	1,414	176 (12.5)	0.20	960	128 (13.4)	0.21
2019	1,067	121 (11.4)	0.18	657	73 (11.3)	0.18

* The smoking prevalence (%) and the mean score were calculated by excluding persons whose smoking status was missing.

†Scores of 0, 1, 2, and 3 were assigned to the number of cigarettes smoked per day of 0, <=10, 11–20, and >=21 cigarettes, respectively.

Supplementary Table 4. Number of participants, smoking prevalence, and the mean score of the number of cigarettes smoked per day by the participants' characteristics among married men

Characteristics	Married men		
	Number of persons	Number of smokers (%)*	Mean score of the number of cigarettes smoked per day *†
Total	5,049	2,042 (41.3)	0.78
Age group			
20–24 years	52	30 (60.0)	1.02
25–29 years	297	129 (44.8)	0.75
30–34 years	749	300 (40.4)	0.71
35–39 years	1,181	478 (41.7)	0.80
40–44 years	1,379	573 (42.4)	0.82
45–49 years	1,391	532 (39.0)	0.77
Marital status			
Married	5,049	2,042 (41.3)	0.78
Never-married	-	-	-
Widowed/divorced	-	-	-
Educational attainment			
Less than high school	215	149 (70.3)	1.50
High school	1,705	836 (50.3)	0.97
Professional training college	647	265 (42.3)	0.76
Technical college/Junior college	168	72 (43.6)	0.87
University	1,826	506 (28.0)	0.50
Currently enrolled in school	29	14 (53.8)	1.12
Missing	459	200 (44.7)	0.85
Employment status			
Regular worker	4,370	1,730 (40.3)	0.75
Non-regular worker	56	26 (48.1)	1.04
Self-employed worker	523	244 (48.4)	0.99
Other workers	25	9 (39.1)	0.87
Unemployed person	63	29 (49.2)	1.02
Missing	12	4 (44.4)	0.75
Household income			
Quantile 1 (Lowest)	903	439 (49.9)	0.95
Quantile 2	1,455	609 (42.9)	0.81
Quantile 3	1,484	582 (39.7)	0.74
Quantile 4 (Highest)	1,207	412 (34.9)	0.68

Residential type			
Owning a house	3,297	1,281 (39.8)	0.76
Rental or employment housing	1,546	657 (43.1)	0.81
Lodging or others	206	104 (52.5)	1.02
Having children aged 0–4 years			
No	3,176	1,319 (42.6)	0.83
Yes	1,873	723 (39.2)	0.71
Missing			
Having children aged 5–9 years			
No	3,126	1,227 (40.0)	0.76
Yes	1,923	815 (43.5)	0.82
Missing			
Having children aged 10–14 years			
No	4,054	1,628 (40.9)	0.77
Yes	995	414 (42.8)	0.82
Missing			
Having children aged 15–19 years			
No	4,074	1,607 (40.3)	0.75
Yes	975	435 (45.7)	0.93
Missing			
Number of children			
None	1,037	396 (39.2)	0.75
One	1,483	576 (39.4)	0.74
Two	1,869	775 (42.4)	0.80
Three or more	660	295 (46.0)	0.88
Missing			
Year			
2010	1,460	616 (44.3)	0.88
2013	1,412	605 (43.3)	0.81
2016	1,240	492 (40.0)	0.75
2019	937	329 (35.6)	0.63

* The smoking prevalence (%) and the mean score were calculated by excluding persons whose smoking status was missing.

†Scores of 0, 1, 2, and 3 were assigned to the number of cigarettes smoked per day of 0, <=10, 11–20, and >=21 cigarettes, respectively.

Supplementary Table 5. Results of the regression analyses investigating associations between the ages of children and smoking prevalence by sex and marital status using multiple imputation

Marital status and the ages of children	Women		Men	
	Adjusted PR (95% CI)*	p-value	Adjusted PR (95% CI)*	p-value
All persons				
Having children aged 0–4 years (Reference: No)	0.62 (0.53, 0.73)	<0.001	0.93 (0.86, 1.00)	0.054
Having children aged 5–9 years (Reference: No)	0.97 (0.85, 1.10)	0.619	1.09 (1.01, 1.16)	0.017
Having children aged 10–14 years (Reference: No)	0.90 (0.77, 1.04)	0.163	0.99 (0.91, 1.08)	0.826
Having children aged 15–19 years (Reference: No)	1.07 (0.93, 1.24)	0.346	1.11 (1.02, 1.22)	0.015
Married persons				
Having children aged 0–4 years (Reference: No)	0.61 (0.51, 0.72)	<0.001	0.92 (0.85, 0.99)	0.026
Having children aged 5–9 years (Reference: No)	0.97 (0.84, 1.12)	0.666	1.11 (1.03, 1.19)	0.004
Having children aged 10–14 years (Reference: No)	0.93 (0.78, 1.10)	0.378	1.01 (0.92, 1.10)	0.868
Having children aged 15–19 years (Reference: No)	1.07 (0.90, 1.27)	0.46	1.15 (1.05, 1.26)	0.003
Unmarried persons				
Having children aged 0–4 years (Reference: No)	0.84 (0.57, 1.25)	0.398	-	-
Having children aged 5–9 years (Reference: No)	0.91 (0.69, 1.20)	0.511	-	-
Having children aged 10–14 years (Reference: No)	0.73 (0.53, 1.01)	0.056	-	-
Having children aged 15–19 years (Reference: No)	1.10 (0.84, 1.45)	0.492	-	-

PR, prevalence ratio; CI, confidence interval
*Age group, household income, educational attainment, marital status, residential type, employment status, and year were adjusted as the explanatory variables, while marital status was not used in the analysis of married persons.

Supplementary Table 6. Results of the regression analyses investigating associations between the number of children and smoking prevalence by sex and marital status using multiple imputation

Marital status and the number of children	Women		Men	
	Adjusted PR (95% CI)*	p-value	Adjusted PR (95% CI)*	p-value
All persons				
None	Reference		Reference	
One	0.88 (0.76, 1.02)	0.097	1.00 (0.91, 1.10)	0.990
Two	0.80 (0.68, 0.93)	0.005	1.07 (0.97, 1.17)	0.177
Three or more	0.77 (0.62, 0.95)	0.017	1.11 (0.99, 1.24)	0.062
Married persons				
None	Reference		Reference	
One	0.83 (0.70, 1.00)	0.045	1.01 (0.92, 1.12)	0.814
Two	0.79 (0.66, 0.94)	0.008	1.09 (0.99, 1.20)	0.080
Three or more	0.73 (0.57, 0.93)	0.009	1.14 (1.02, 1.28)	0.024
Unmarried persons				
None	Reference		-	-
One	1.01 (0.75, 1.37)	0.931	-	-
Two	0.81 (0.57, 1.14)	0.221	-	-
Three or more	1.02 (0.60, 1.75)	0.934	-	-

PR, prevalence ratio; CI, confidence interval

*Age group, household income, educational attainment, marital status, residential type, employment status, and year were adjusted as the explanatory variables, while marital status was not used in the analysis of married persons.

Supplementary Table 7. Results of the regression analyses investigating associations between the ages of children and the number of cigarettes smoked per day by sex and marital status using multiple imputation

Marital status and the ages of children	Women		Men	
	Adjusted OR (95% CI)*	p-value	Adjusted OR (95% CI)*	p-value
All persons				
Having children aged 0–4 years (Reference: No)	0.55 (0.46, 0.67)	<0.001	0.84 (0.74, 0.96)	0.011
Having children aged 5–9 years (Reference: No)	0.94 (0.80, 1.10)	0.421	1.13 (1.01, 1.28)	0.04
Having children aged 10–14 years (Reference: No)	0.87 (0.72, 1.05)	0.158	0.95 (0.82, 1.11)	0.54
Having children aged 15–19 years (Reference: No)	1.11 (0.92, 1.34)	0.27	1.28 (1.09, 1.50)	0.002
Married persons				
Having children aged 0–4 years (Reference: No)	0.53 (0.43, 0.66)	<0.001	0.83 (0.73, 0.95)	0.007
Having children aged 5–9 years (Reference: No)	0.95 (0.79, 1.13)	0.562	1.16 (1.03, 1.32)	0.016
Having children aged 10–14 years (Reference: No)	0.91 (0.74, 1.12)	0.359	0.97 (0.83, 1.14)	0.74
Having children aged 15–19 years (Reference: No)	1.11 (0.90, 1.36)	0.346	1.34 (1.14, 1.59)	<0.001
Unmarried persons				
Having children aged 0–4 years (Reference: No)	0.81 (0.45, 1.46)	0.491	-	-
Having children aged 5–9 years (Reference: No)	0.79 (0.51, 1.22)	0.286	-	-
Having children aged 10–14 years (Reference: No)	0.63 (0.39, 1.00)	0.051	-	-
Having children aged 15–19 years (Reference: No)	1.15 (0.76, 1.73)	0.514	-	-

OR, odds ratio; CI, confidence interval

*Age group, household income, educational attainment, marital status, residential type, employment status, and year were adjusted as the explanatory variables, while marital status was not used in the analysis of married persons.

Supplementary Table 8. Results of the regression analyses investigating associations between the number of children and the number of cigarettes smoked per day by sex and marital status using multiple imputation

Marital status and the number of children	Women		Men	
	Adjusted OR (95% CI)*	p-value	Adjusted OR (95% CI)*	p-value
All persons				
None	Reference		Reference	
One	0.85 (0.70, 1.03)	0.097	0.96 (0.82, 1.12)	0.588
Two	0.73 (0.60, 0.90)	0.002	1.07 (0.91, 1.25)	0.409
Three or more	0.69 (0.53, 0.91)	0.007	1.15 (0.94, 1.40)	0.168
Married persons				
None	Reference		Reference	
One	0.79 (0.64, 0.99)	0.039	0.98 (0.83, 1.15)	0.801
Two	0.73 (0.59, 0.91)	0.006	1.10 (0.93, 1.29)	0.257
Three or more	0.66 (0.49, 0.88)	0.004	1.19 (0.97, 1.45)	0.098
Unmarried persons				
None	Reference		-	-
One	1.02 (0.65, 1.60)	0.935	-	-
Two	0.64 (0.38, 1.07)	0.086	-	-
Three or more	0.94 (0.42, 2.13)	0.885	-	-

OR, odds ratio; CI, confidence interval

*Age group, household income, educational attainment, marital status, residential type, employment status, and year were adjusted as the explanatory variables, while marital status was not used in the analysis of married persons.
